# Mobile Prenatal Education and Its Impact on Reducing Adverse Pregnancy Outcomes: Retrospective Real-World Study

**DOI:** 10.2196/46910

**Published:** 2023-12-20

**Authors:** Jie Hao, Lin Yang, Yaxin Wang, Yushan Lan, Xiaowei Xu, Ziyang Wang, Zanmei Li, Liangkun Ma, Jiao Li, Suhan Zhang, Yin Sun

**Affiliations:** 1 Institute of Medical Information & Library Chinese Academy of Medical Sciences & Peking Union Medical College Beijing China; 2 Department of Obstetrics and Gynecology Peking Union Medical College Hospital Beijing China

**Keywords:** adverse pregnancy outcome, mobile prenatal education, pregnancy, real-world study, retrospective study

## Abstract

**Background:**

Pregnancy is a pivotal phase in a woman’s life, demanding special attention to ensure maternal and fetal health. Prenatal education plays a vital role in promoting healthy pregnancies and reducing adverse outcomes for pregnant women. Mobile prenatal education programs have gained traction due to their accessibility and timeliness, especially in light of finite health care resources and the constraints imposed by the COVID-19 pandemic.

**Objective:**

This study aims to develop and evaluate the effectiveness of a mobile-based prenatal education program in improving pregnancy outcomes.

**Methods:**

We developed a mobile-based prenatal education curriculum in collaboration with a multidisciplinary maternal care team from Peking Union Medical College Hospital (PUMCH) in Beijing, China. Data were retrospectively collected from 1941 pregnant women who had registered for the PUMCH mobile prenatal education program and subsequently delivered at PUMCH between May 2021 and August 2022. The study compared pregnancy outcomes between the completing group, which were pregnant women who had completed at least 1 course, and the noncompleting group. We also analyzed differences among course topics within the completing group and assessed course topic popularity among pregnant women.

**Results:**

The PUMCH mobile prenatal education curriculum consists of 436 courses across 9 topics. Out of the participants, a total of 1521 did not complete any courses, while 420 completed at least 1 course. Compared with the noncompleting group, pregnant women who completed courses exhibited a significant reduction in the risk of gestational diabetes mellitus, induced abortion, postpartum infection, fetal intrauterine distress, and neonatal malformation. Among those in the completing group, a total of 86% (361/420) started course completion during the first and second trimesters. Furthermore, completing courses related to topics of pregnancy psychology and pregnancy nutrition was associated with reduced risks of premature rupture of membranes and small for gestational age infants, respectively. Pregnancy psychology and postpartum recovery were the preferred topics among pregnant women.

**Conclusions:**

The study demonstrates the potential of mobile-based prenatal education programs in improving pregnancy outcomes and supporting health care providers in delivering effective prenatal education. The rise of mobile prenatal education presents an opportunity to improve maternal and child health outcomes. Further research and broader implementation of such programs are warranted to continually improve maternal and child health.

## Introduction

Pregnancy brings about a multitude of psychological and social changes in a woman’s life [1]. Access to reliable information can aid pregnant women in making informed and healthier decisions [2]. Therefore, it is important to recognize pregnant women as the primary target audience for prenatal education, aiming to mitigate potential adverse outcomes [3]. To achieve desirable pregnancy outcomes, it is essential that pregnant women receive comprehensive and high-quality prenatal education.

Prenatal education has been widely acknowledged for its ability to increase prenatal examination usage [4], enhance the psychological well-being of expectant mothers [5], and contribute to improved delivery outcomes [6], among other benefits. Recent studies have highlighted the beneficial effects of prenatal education, particularly for women experiencing their first pregnancy. A retrospective analysis demonstrated that those who participated in childbirth classes were more likely to achieve successful normal vaginal deliveries, contributing positively to overall pregnancy outcomes [1]. Furthermore, the effectiveness of psychoeducational interventions has been confirmed through a randomized control trial, effectively reducing the fear of childbirth among anxious pregnant women [7]. Similarly, simulation-based childbirth education programs have demonstrated their capacity to alleviate the fear of childbirth among Chinese primiparas, and in high-income countries, childbirth training workshops have effectively reduced the incidence of unnecessary cesarean sections [8]. In another randomized controlled trial, a combination of 2 in-person and 11 telephone sessions focused on promoting healthy pregnancy behaviors led to a reduction in the weekly rate of gestational weight gain in pregnant women [9].

While traditional group prenatal education has been traditionally conducted in face-to-face settings, thereby limiting participant numbers, the evolving health care landscape has introduced new challenges. The increasing burden on health care systems has made it challenging for medical professionals to provide personalized health education to every expectant mother using conventional methods. In response to these accessibility issues and to better cater to the evolving needs of pregnant women and their partners, web-based prenatal education has gained significant traction [10]. Web-based education represents a novel and efficient teaching method, offering a more effective and timely approach compared to traditional face-to-face education [11]. During the COVID-19 pandemic and amid rising health care costs, the popularity of web-based education has soared [12,13]. A randomized clinical trial conducted among low-income postpartum women demonstrated that web-based education could reduce maternal weight gain during pregnancy [14]. Additionally, researchers provided web-based articles on physical activity during pregnancy and observed improvements in pregnant women’s physical activity levels [15]. Furthermore, a randomized controlled trial highlighted the benefits of web-based prenatal education, including a reduction in concerns about labor, fear of childbirth, and fear of COVID-19 during the pandemic [16].

Mobile-based learning has also emerged as a promising approach for supporting prenatal education [17]. A smartphone-based prenatal education program aimed at parents at risk of preterm birth showcased its ability to raise awareness about preterm birth and establish a foundational knowledge base for making informed medical care choices [18]. In China, mobile-based prenatal education has gained significant traction [19], with thousands of maternal health-related apps available, most of which focus on prenatal education [20]. Recent statistics reveal that approximately half of the pregnant women in China have used maternal-related apps [21], with the majority participating in at least 1 mobile-based prenatal course during their early and mid-pregnancy [22]. Despite this, both national surveys have highlighted a shared desire among pregnant women for the development of evidence-based and well-informed mobile-based prenatal education programs, endorsed by obstetricians [21,22]. The needs of pregnant women underscore the prevailing tendency of existing mobile-based prenatal education programs to narrow their focus on specific topics, often lacking the comprehensive curriculum design offered by experienced obstetrician-led teams.

In this study, we present a comprehensive mobile-based prenatal education program tailored to the needs of pregnant women. Deployed within a hospital-authorized smartphone app, our program seeks to enhance the accessibility and cost-effectiveness of prenatal education. To evaluate the program’s effectiveness, we conducted a retrospective real-world analysis of app module usage records and clinical outcomes among pregnant women. Our mobile-based prenatal education module helps to address the need for professional health care support and improve outcomes for expectant mothers.

## Methods

**Study Design**

**Figure 1:**
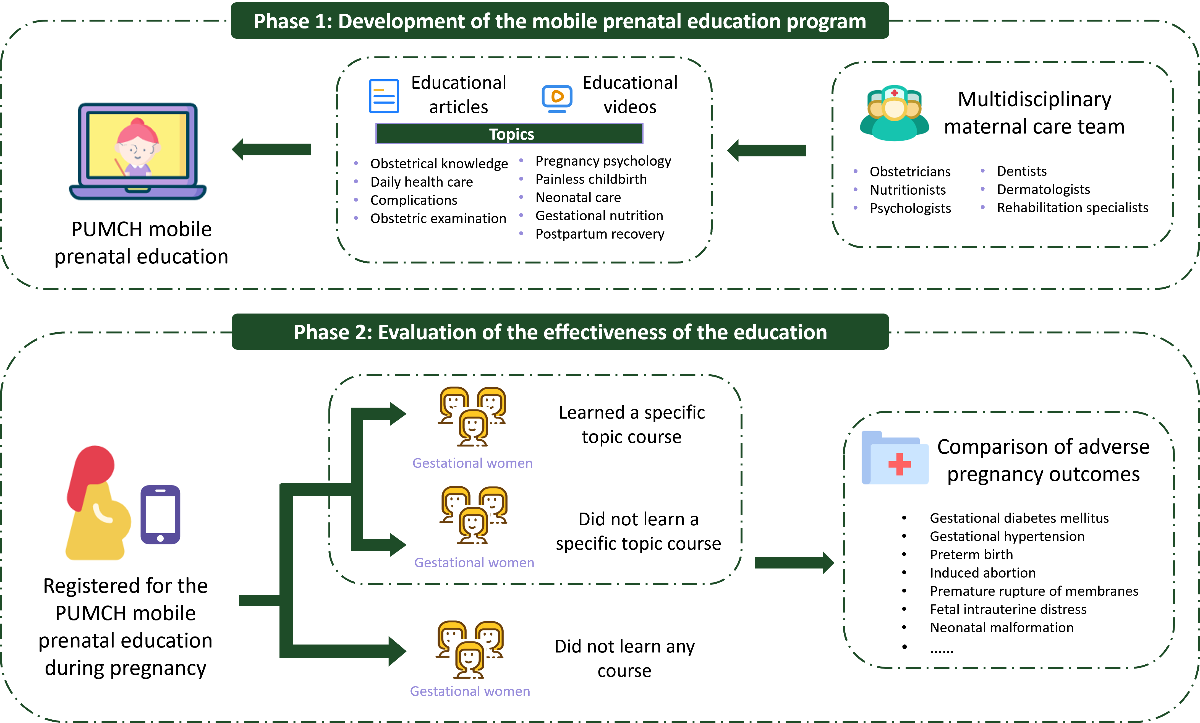
Workflow of developing and evaluating the Peking Union Medical College Hospital (PUMCH) mobile prenatal education.

This study consists of 2 distinct phases. In the first phase, we developed a comprehensive curriculum for a mobile-based prenatal education program with the invaluable support and expertise of a multidisciplinary maternal care team at the Peking Union Medical College Hospital (PUMCH) in Beijing, China. All courses were designed to be easily accessible using smartphones. The second phase involved evaluating the effectiveness of our prenatal education program by analyzing the records of class attendance. The workflow of this study is shown in Figure 1.

### Development of the PUMCH Mobile Prenatal Education

The curriculum of our mobile-based prenatal education was designed and implemented by a multidisciplinary maternal care team at the PUMCH in Beijing, China. This team includes obstetricians, nutritionists, psychologists, dentists, dermatologists, and rehabilitation specialists, all possessing extensive expertise with over 20 years of traditional prenatal education. Drawing upon evidence-based insights gathered from pregnant women [21,22], the PUMCH team developed a comprehensive curriculum comprising a total of 436 courses, organized into 9 topics, which are obstetrical knowledge, gestational nutrition, daily health care, complications, obstetric examination, pregnancy psychology, painless childbirth, neonatal care, and postpartum recovery.

To optimize the learning experience, the courses were structured to span the entire duration of pregnancy. Roughly 1-2 courses were made available for each topic daily, and each course required just about 5 minutes to complete. This scheduling accommodated the busy routines of pregnant women, enabling them to learn effectively during shorter, fragmented periods of time. Furthermore, 2 external nurses conducted individual reviews of the developed courses to maintain the quality of our content. To enhance the knowledge acquisition experience, these courses were presented in a multimedia format, including articles and videos.

In addition, all courses were seamlessly integrated into the official PUMCH app, which provides a wide range of patient services, including registration, payment, and health education. Upon visiting the obstetrician and completing the registration process, pregnant women were granted authorized access to the PUMCH mobile prenatal education curriculum through this official app. This convenient platform allowed pregnant women to access comprehensive prenatal education at their convenience, free from the constraints of location and time that are often associated with traditional prenatal programs.

Recognizing that different trimesters of pregnancy require specific knowledge, we automated the course recommendations for pregnant women based on their current pregnancy trimester. Throughout the pregnancy journey, these tailored course recommendations were delivered daily, ensuring that expectant mothers received knowledge precisely suited to their specific stage of pregnancy.

### Evaluation of the Effectiveness of the PUMCH Mobile Prenatal Education

To evaluate the effectiveness of the PUMCH mobile prenatal education program, we conducted multiple comparisons to assess its impacts on the pregnancy outcomes of registered pregnant women. This evaluation aimed to (1) determine whether completing courses can reduce the occurrence of adverse pregnancy outcomes in comparison to not participating in any courses, and (2) examine the impact of learning specific topics on pregnancy outcomes among pregnant women who completed the courses.

#### Study Population

Through the official PUMCH app, we retrospectively collected records from 1941 pregnant women who had registered for the PUMCH mobile prenatal education curriculum and subsequently delivered at the PUMCH in Beijing, China, during the period from May 2021 to August 2022. The inclusion criteria included singleton pregnancy and registration in the PUMCH mobile prenatal education program before delivery. The exclusion criteria were as follows: (1) diabetes mellitus (DM), (2) hypertension, and (3) a history of smoking [23].

#### Data Collection

Course-taking records, including details such as course titles, topics, and timings, were retrieved through the app from all eligible pregnant women participating in this study. Additionally, maternal characteristics and adverse pregnancy outcomes were extracted from electronic health records. Maternal characteristics included maternal age, prepregnancy BMI, parity, gravidity, history of abortion, history of gestational diabetes mellitus (GDM), history of abnormal pregnancy, family history of DM, family history of hypertension, and polycystic ovary syndrome (PCOS). Adverse pregnancy outcomes included GDM, gestational hypertension, postpartum infection, preterm birth, induced abortion, premature rupture of membranes (PROM), fetal intrauterine distress, and neonatal malformation.

#### Statistical Analyses

The data were analyzed with Python (version 3.8.8; Python Software Foundation). As for the maternal characteristics and adverse pregnancy outcomes, continuous variables were reported as mean (SD) and categorical variables as n (%). To determine the effectiveness of the PUMCH mobile prenatal education, the 2-tailed *t* test was performed for continuous variables and the chi-square test for categorical variables, and *P*<.05 was considered statistically significant. In addition, multiple logistic regressions, adjusted by maternal characteristics, were performed.

### Ethical Considerations

As we used anonymized and deidentified data and did not constitute human research, the need for written informed consent was waived by the Ethics Review Board of the PUMCH due to the retrospective nature of the study. This study was approved by the Ethics Review Board of the PUMCH (I-22PJ122). All procedures were performed in accordance with the Declaration of Helsinki.

## Results

### The PUMCH Mobile Prenatal Education

In this study, we implemented a total of 436 mobile-based prenatal courses with valuable support from the PUMCH multidisciplinary maternal care team. These courses included 234 educational videos and 202 articles. An illustrative example of course titles and formats for 3 topics is presented in Table 1.

**Table 1:** Example of course titles and formats for 3 topics in the Peking Union Medical College Hospital (PUMCH) mobile prenatal education program.

Topic and title		Format
**Obstetrical knowledge**		
	How to distinguish between true and false labor contractions?	Video
	How to determine if a vaginal delivery is possible?	Video
	Is vaginal bleeding in late pregnancy always a sign of labor onset?	Article
	Don't be misled again! Common pregnancy myths.	Article
**Gestational nutrition**		
	Is eating pork liver effective for treating anemia during pregnancy?	Video
	When encountering gestational diabetes, how should expectant mothers adjust their diet?	Video
	Dietary adjustments in early pregnancy	Article
	How to supplement iron?	Article
**Daily health care**		
	Pregnant and have a cold? Be cautious about avoiding medication.	Video
	Four ways to relieve abdominal and lower back pain during pregnancy.	Video
	Precautions for exercise during pregnancy.	Article
	How to perform chest and upper arm stretches during pregnancy?	Article

**Figure 2:**
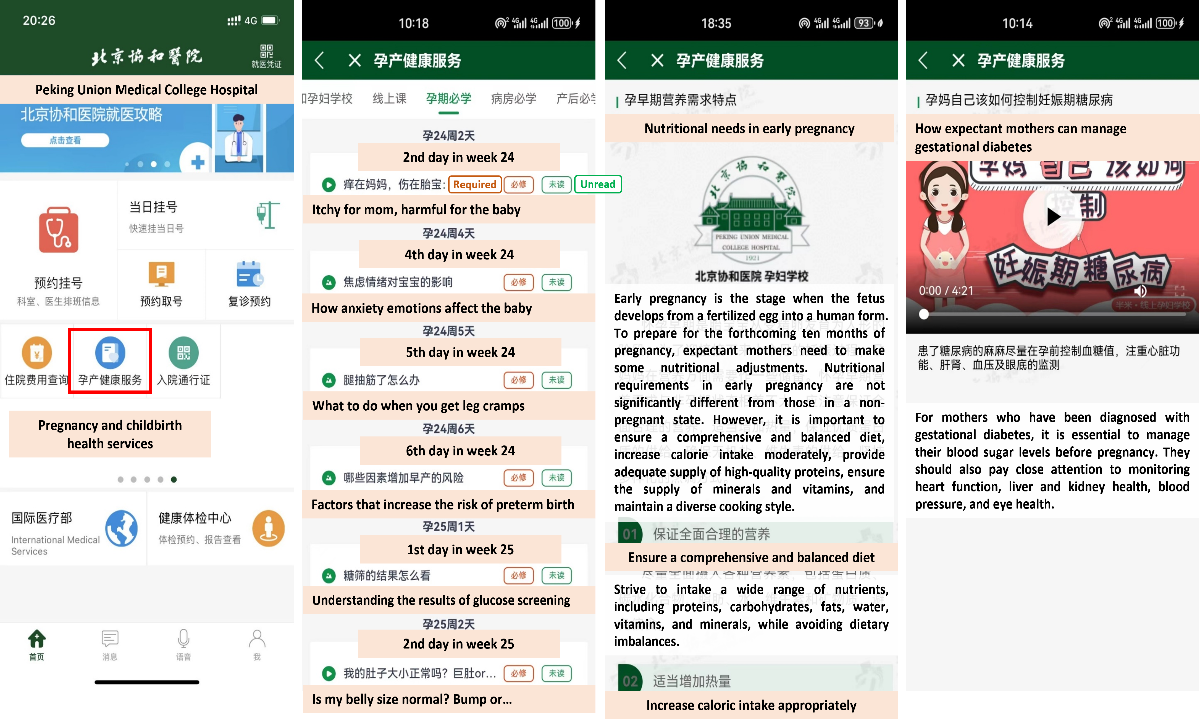
Snapshots of the Peking Union Medical College Hospital (PUMCH) mobile prenatal education program. From left to right: the main page of the official PUMCH app, recommended prenatal courses organized by the number of days, a course in article format, and a course in video format.

These courses were accessible to pregnant women through the official PUMCH app. In addition to receiving daily course recommendations tailored to their trimesters, pregnant women had the flexibility to explore other courses of interest, allowing them to acquire prenatal knowledge according to their individual needs. Figure 2 provides snapshots of the PUMCH mobile prenatal education interface. The first snapshot reveals the easy accessibility of our program on the main page of the official app, seamlessly integrated with other medical services. The second snapshot highlights the prenatal course recommended to pregnant women, taking into account their specific pregnancy stage in terms of the number of days. As illustrated in the third and fourth snapshots, all courses were available in either article or video format.

### Participants Characteristics

A total of 1941 pregnant women were included in the study, with 1521 of them not completing any courses, while 420 completed at least 1 course. Table 2 presents the maternal characteristics of participants in these 2 groups. The average age of participants in both groups is approximately 32 years. In the group that did not complete the courses, the proportion of participants of advanced maternal age (35 years of age or older) is 25.05% (381/1521), which is about 3% higher than the group that completed courses. There is a statistically significant difference in prepregnancy BMI values (kg/m^2^) between the 2 groups (*P*=.04), with participants in the completing group having higher BMI values than those in the not completing group. Additionally, parity and gravidity also exhibit significant differences between the 2 groups with very small *P* values (*P*<.001). In the completing group, participants have a higher proportion of nullipara, primigravida, or both.

**Table 2:** Maternal characteristics of participants between 2 groups of not completing and completing courses.

Characteristics			Noncompleting (n=1521)		Completing (n=420)	*P* value	
Maternal age (years), mean (SD)			32.01 (3.83)		32.06 (3.69)	.80	
**Maternal age group (years), n (%)**							.18
	<35	1140 (74.95)		328 (78.10			
	≥35	381 (25.05)		92 (21.90)			
Prepregnancy BMI (kg/m^2^), mean (SD)			23.72 (3.41)		24.12 (3.39)	*.04* ^a^	
**BMI group (kg/m^2^), n (%)**							.17
	<18.5	39 (2.56)		7 (1.66)			
	18.5-24	816 (53.65)		206 (49.05)			
	24-28	498 (32.74)		159 (37.86)			
	≥28	168 (11.05)		48 (11.43)			
**Parity, n (%)**							*<.001*
	Nullipara	1179 (77.51)		375 (89.29)			
	Multipara	342 (22.49)		45 (10.71)			
**Gravidity, n (%)**							*<.001*
	Primigravid	876 (57.59)		278 (66.19)			
	Multigravid	645 (42.41)		142 (33.81)			
**History of abortion, n (%)**							.79
	No	1069 (70.28)		298 (70.95)			
	Yes	452 (29.72)		122 (29.05)			
**History of GDM^b^, n (%)**							.53
	No	1514 (99.54)		419 (99.76)			
	Yes	7 (0.46)		1 (0.24)			
**History of abnormal pregnancy, n (%)**							.22
	No	1445 (95)		405 (96.43)			
	Yes	76 (5)		15 (3.57)			
**PCOS^c^, n (%)**							.24
	No	1461 (96.06)		398 (94.76)			
	Yes	60 (3.94)		22 (5.24)			
**Family history of DM^d^, n (%)**							.53
	No	1203 (79.09)		338 (80.48)			
	Yes	318 (20.91)		82 (19.52)			
**Family history of hypertension, n (%)**							.06
	No	1012 (66.54)		300 (71.43)			
	Yes	509 (33.46)		120 (28.57)			

^a^Italics indicate significant values.

^b^GDM: gestational diabetes mellitus.

^c^PCOS: polycystic ovary syndrome.

^d^DM: diabetes mellitus.

### Statistics on Completing the PUMCH Mobile Prenatal Courses

In the PUMCH mobile prenatal education program, we have a total of 436 courses available, with 234 presented in video format and 202 in article format. These courses are categorized into 1 topic, and the topic with the highest number of courses is obstetrical knowledge, accounting for approximately 24.5% (107/436) of the total course count. Among the 420 pregnant women who completed at least 1 course, we observed that 43.8% (184/420) of them began completing courses during their first trimester, and a significant 86% (361/420) started course completion during their first and second trimesters. Table 3 shows that the topics of obstetrical knowledge and daily health care were the most popular, with completion rates over 80% (351/420 and 344/420). Furthermore, when considering the average number of participants per course, it was obvious that the topics of pregnancy psychology and postpartum recovery had the most attention. Detailed participant counts for each topic are provided in Table 3.

**Table 3:** Statistics on the Peking Union Medical College Hospital (PUMCH) mobile prenatal education: topics, course count (%), participant count (%), and average participants per course.

Topics	Courses, n (%)	Participants who completed courses (n=420), n (%)	Average number of participants per course
Obstetrical knowledge	107 (24.5)	351 (83.6)	3.28
Daily health care	73 (16.7)	344 (81.9)	4.71
Complications	71 (16.3)	244 (58.1)	3.43
Gestational nutrition	44 (10.1)	295 (70.2)	6.70
Pregnancy psychology	34 (7.8)	278 (66.2)	8.17
Obstetric examination	33 (7.6)	234 (55.7)	7.09
Neonatal care	29 (6.7)	150 (35.7)	5.17
Painless childbirth	25 (5.7)	171 (40.7)	6.84
Postpartum recovery	20 (4.6)	143 (34)	7.15

### Comparison of Adverse Pregnancy Outcomes Between the Noncompleting and Completing Groups

Table 4 displays the comparisons of adverse pregnancy outcomes between the 2 groups. There are statistically significant differences in adverse pregnancy outcomes for GDM, postpartum infection, induced abortion, fetal intrauterine distress, and neonatal malformation between the 2 groups. For instance, the odds ratio (OR) value for the noncompleting group compared with the completing group in GDM is 0.3043, which means that the odds of GDM in the completing group are approximately 30% lower than the odds in the noncompleting group. This significant OR value indicates that participants in the completing group have a lower risk of GDM compared with noncompleting group. Except for neonatal malformation, for which there were no cases in the completing group, multiple logistic regressions were conducted for each significant adverse pregnancy outcome, adjusted by maternal age group, BMI group, parity, and gravidity. GDM, postpartum infection, and fetal intrauterine distress remained statistically significant. Detailed results are provided in Table S1 in Multimedia Appendix 1. However, there were no significant differences observed between the 2 groups for gestational hypertension, preterm birth, PROM, macrosomia, and small for gestational age (SGA).

**Table 4:** Adverse pregnancy outcomes of participants between the noncompleting and completing groups.

Adverse pregnancy outcomes	Noncompleting (n=1521), n (%)	Completing (n=420), n (%)	*P* value	OR^a^
GDM^b^	285 (18.74)	98 (23.33)	*.04* ^c^	0.3043
Gestational hypertension	30 (1.97)	8 (1.90)	.93	0.0194
Postpartum infection	115 (7.56)	52 (12.38)	*.002*	0.1413
Induced abortion	419 (27.55)	143 (34.05)	*.009*	0.5162
Preterm birth	98 (6.44)	23 (5.47)	.47	0.0579
PROM^d^	391 (25.71)	126 (30)	.08	0.4286
Fetal intrauterine distress	219 (14.40)	83 (19.76)	*.007*	0.2463
Neonatal malformation	17 (1.11)	0 (0)	*.03*	0.0000
SGA^e^	12 (0.79)	4 (0.95)	.74	0.0096
Macrosomia	67 (4.41)	15 (3.57)	.45	0.0370

^a^OR: odds ratio.

^b^GDM: gestational diabetes mellitus.

^c^Italics indicate significant values.

^d^PROM: premature rupture of membranes.

^e^SGA: small for gestational age.

### Comparison of Adverse Pregnancy Outcomes Among Course Topics in the Completing Group

We examined whether completing specific course topics reduced adverse pregnancy outcomes among participants in the completing group. For each topic, participants were categorized into 2 groups: those who completed the topic and those who did not complete it. Table 5 presents the significant differences in adverse pregnancy outcomes between the “completing the topic” group and the “not completing the topic” group. Participants who completed the pregnancy psychology topic course experienced a reduced risk of PROM compared with the “not completing the topic” group (*P*=.03 and OR=0.5027). Additional results for pairs of topics and adverse pregnancy outcomes are available in Table S2 in Multimedia Appendix 1.

**Table 5:** Effectiveness of adverse pregnancy outcomes among participants in the completing group for different topics.

Adverse pregnancy outcomes	Topics	Not completing the topic, n/N (%)	Completing the topic, n/N (%)	*P* value	OR^a^
PROM^b^	Pregnancy psychology	33/142 (25.71)	93/278 (30)	*.03* ^c^	0.5027
SGA^d^	Gestational nutrition	3/125 (0.79)	1/295 (0.95)	*.05*	0.0034

^a^OR: odds ratio.

^b^PROM: premature rupture of membranes.

^c^Italics indicate significant values.

^d^SGA: small for gestational age.

## Discussion

### Principal Findings

In this study, we have collaboratively designed a mobile-based prenatal education program with the PUMCH multidisciplinary maternal care team. This mobile-based curriculum offers 436 courses across 9 topics and is accessible through the official PUMCH app, providing pregnant women with valuable guidance throughout their entire pregnancy journey. A mixed methods study has indicated that web-based prenatal education can positively influence lifestyle choices and the ease of accessing health information during pregnancy [21]. The COVID-19 pandemic has heightened the importance of easily accessible health care resources, especially for pregnant women at increased risk of severe illness if infected [24]. The World Health Organization (WHO) advises pregnant women to avoid crowded and poorly ventilated indoor spaces to reduce the risk of COVID-19 transmission. Therefore, there is an urgent need for cost-effective and easily accessible mobile-based prenatal education programs to support expectant mothers, especially in these challenging times.

To evaluate the effectiveness of our mobile-based program, we compared adverse pregnancy outcomes between the group that completed courses and the group that registered but did not complete any courses. Our data, derived from real-world app records, revealed an imbalance between the 2 groups, with approximately 21.6% (420/1941) of registered pregnant women completing at least 1 course. Significantly, pregnant women who completed courses demonstrated a reduced risk of adverse pregnancy outcomes such as GDM, postpartum infection, induced abortion, fetal intrauterine distress, and neonatal malformation. For instance, GDM, a common pregnancy complication, is associated with the risk of hyperglycemia and adverse short- and long-term health outcomes for both mothers and infants [25]. Our curriculum addresses GDM directly, offering courses on improving physical activity and nutrition, potentially lowering the risk of GDM. Taking those courses shows the potential to lower the risk of GDM by delivering knowledge about improving physical activity, nutrition intake, etc [26,27].

Furthermore, we analyzed the behavior of pregnant women who completed specific topic courses in the completing group. While the impact of completing the specific topic courses on reducing adverse pregnancy outcomes was generally modest, we identified 2 significant findings. First, completing the pregnancy psychology course was associated with a statistically significant reduction in the risk of PROM, and second, completing the pregnancy nutrition course may prevent the occurrence of SGA infants.

Nutrition during pregnancy plays an important role in lifelong health [28], especially in terms of brain development and behavior [29]. As many adverse birth outcomes originate during pregnancy [30], awareness of nutritional balance and healthy dietary habits can reduce the risk of outcomes like SGA. SGA, typically defined as birthweight below the tenth percentile [31], is a known risk factor for stillbirth [32]. Previous research has demonstrated that nutrition interventions and adopting healthy dietary patterns can significantly lower the risk of SGA [33]. Moreover, maternal creatine supplementation during pregnancy has shown the potential to reduce the risk of neonatal asphyxia, as evidenced in small animal studies [34].

We observed that 86% (361/420) of participants started course completion during their first and second trimesters, in line with survey results on Chinese web-based prenatal education [22]. Pregnancy psychology and postpartum recovery are the most popular topics. These findings can inform the development of additional educational courses on both topics and further engage pregnant women.

Our mobile-based prenatal education program serves as a valuable source of information, enhancing pregnant women’s knowledge regarding gestational nutrition, recommended practices, and anticipated challenges. Given that pregnant women increasingly seek information on the web [35], our program not only meets the rising demand for accessible mobile prenatal education but also bridges a significant gap in this field by offering a cost-effective and efficient source of information [22,35]. By promoting this curriculum more widely, we aim to encourage healthier lifestyles and enhance pregnancy outcomes.

### Limitations

This study has several limitations. First, the effectiveness of our mobile-based prenatal education program was assessed among pregnant women from a single center, specifically PUMCH in Beijing, and its generalizability to other settings needs to be confirmed. Second, the analysis was based on 1 year of data, and a larger sample size with a longer duration would provide more robust estimates of the effectiveness of our mobile-based program. Third, as this is a retrospective study with outcomes and information collected post pregnancy, potential confounders and biases may have influenced the results. To establish the effectiveness of mobile-based prenatal education more conclusively, future research should consider conducting randomized controlled trials or prospective cohort studies.

### Conclusion

This study outlined the development of a mobile-based prenatal education program and assessed its effectiveness by analyzing adverse pregnancy outcomes among pregnant women. The findings indicate that the mobile-based prenatal education curriculum has the potential to reduce adverse outcomes in pregnant women, offering valuable support for health care providers in delivering effective prenatal education services. The growing prominence of mobile prenatal education presents an opportunity to enhance pregnancy outcomes and the health of both mothers and children.

## Appendix

Multimedia Appendix 1 Result of all comparisons on adverse pregnancy outcomes.

## Abbreviations

DM: diabetes mellitus
GDM: gestational diabetes mellitus
PCOS: polycystic ovary syndrome
PROM: premature rupture of membranes
PUMCH: Peking Union Medical College Hospital
SGA: small for gestational age
WHO: World Health Organization
