# Supplementary material for: Mobile Prenatal Education and Its Impact on Reducing Adverse Pregnancy Outcomes: Retrospective Real-World Study
Source: JMIR Mhealth Uhealth. 2023 Dec 20;11:e46910. doi: 10.2196/46910 (PMC10765290; doi:10.2196/46910)
Supplement: Multimedia Appendix 1 [file mhealth_v11i1e46910_app1.docx]

**Multimedia Appendix 1: Result of all comparisons on adverse pregnancy outcomes**

**Table S1.** Results of multiple logistic regressions for gestational diabetes mellitus (GDM), postpartum infection, induced abortion, and fetal intrauterine distress, adjusted by maternal age group, BMI group, parity, and gravidity.

| Adverse pregnancy outcomes | Coefficient | Standard Error | *P*-values | OR |
| --- | --- | --- | --- | --- |
| GDM | -0.6111 | 0.124 | <0.0001* | 0.5427 |
| Postpartum infection | -1.0762 | 0.158 | <0.0001* | 0.3408 |
| Induced abortion | -0.2105 | 0.112 | 0.060 | 0.8101 |
| Fetal intrauterine distress | -0.6853 | 0.132 | <0.0001* | 0.5039 |

**Table S2.** Comparisons of adverse pregnancy outcomes among participants in the completing group for different topics.

| Topic | Adverse pregnancy outcome | P-values |
| --- | --- | --- |
| Postpartum recovery | GDM | 0.2877 |
|  | Gestational hypertension | 0.1940 |
|  | Postpartum infection | 0.1793 |
|  | Induced abortion | 0.0708 |
|  | Preterm birth | 0.9390 |
|  | PROM | 0.8397 |
|  | Fetal intrauterine distress | 0.3334 |
|  | SGA | 0.1487 |
|  | Macrosomia | 0.9525 |
| Obstetrical knowledge | GDM | 0.9751 |
|  | Gestational hypertension | 0.7620 |
|  | Postpartum infection | 0.8549 |
|  | Induced abortion | 0.8910 |
|  | Preterm birth | 0.6522 |
|  | PROM | 0.6251 |
|  | Fetal intrauterine distress | 0.3834 |
|  | SGA | 0.6420 |
|  | Macrosomia | 0.7418 |
| Obstetric examination | GDM | 0.8891 |
|  | Gestational hypertension | 0.0774 |
|  | Postpartum infection | 0.3663 |
|  | Induced abortion | 0.4901 |
|  | Preterm birth | 0.0707 |
|  | PROM | 0.0945 |
|  | Fetal intrauterine distress | 0.7591 |
|  | SGA | 0.8171 |
|  | Macrosomia | 0.7336 |
| Pregnancy psychology | GDM | 0.7822 |
|  | Gestational hypertension | 0.5948 |
|  | Postpartum infection | 0.4189 |
|  | Induced abortion | 0.3439 |
|  | Preterm birth | 0.4206 |
|  | PROM | 0.0307 |
|  | Fetal intrauterine distress | 0.2927 |
|  | SGA | 0.7082 |
|  | Macrosomia | 0.5514 |
| Gestational nutrition | GDM | 0.4242 |
|  | Gestational hypertension | 0.2062 |
|  | Postpartum infection | 0.6214 |
|  | Induced abortion | 0.2105 |
|  | Preterm birth | 0.9421 |
|  | PROM | 0.9073 |
|  | Fetal intrauterine distress | 0.8506 |
|  | SGA | 0.0467 |
|  | Macrosomia | 0.1564 |
| Neonatal care | GDM | 0.3355 |
|  | Gestational hypertension | 0.1664 |
|  | Postpartum infection | 0.2891 |
|  | Induced abortion | 0.3985 |
|  | Preterm birth | 0.3216 |
|  | PROM | 0.5049 |
|  | Fetal intrauterine distress | 0.9272 |
|  | SGA | 0.6531 |
|  | Macrosomia | 0.8446 |
| Complications | GDM | 0.3416 |
|  | Gestational hypertension | 0.6393 |
|  | Postpartum infection | 0.2550 |
|  | Induced abortion | 0.9873 |
|  | Preterm birth | 0.4764 |
|  | PROM | 0.3002 |
|  | Fetal intrauterine distress | 0.5815 |
|  | SGA | 0.7416 |
|  | Macrosomia | 0.4932 |
| Painless childbirth | GDM | 0.8326 |
|  | Gestational hypertension | 0.3610 |
|  | Postpartum infection | 0.9587 |
|  | Induced abortion | 0.5603 |
|  | Preterm birth | 0.8736 |
|  | PROM | 0.7125 |
|  | Fetal intrauterine distress | 0.5819 |
|  | SGA | 0.5203 |
|  | Macrosomia | 0.5534 |
| Daily health care | GDM | 0.8260 |
|  | Gestational hypertension | 0.6781 |
|  | Postpartum infection | 0.5875 |
|  | Induced abortion | 0.4417 |
|  | Preterm birth | 0.5174 |
|  | PROM | 0.1843 |
|  | Fetal intrauterine distress | 0.7456 |
|  | SGA | 0.0958 |
|  | Macrosomia | 0.6256 |
